# A strain of *Saccharomyces cerevisiae *evolved for fermentation of lignocellulosic biomass displays improved growth and fermentative ability in high solids concentrations and in the presence of inhibitory compounds

**DOI:** 10.1186/1754-6834-4-49

**Published:** 2011-11-10

**Authors:** Gary M Hawkins, Joy Doran-Peterson

**Affiliations:** 1 Microbiology Department, University of Georgia, Athens, GA 30602, USA

## Abstract

**Background:**

Softwoods are the dominant source of lignocellulosic biomass in the northern hemisphere, and have been investigated worldwide as a renewable substrate for cellulosic ethanol production. One challenge to using softwoods, which is particularly acute with pine, is that the pretreatment process produces inhibitory compounds detrimental to the growth and metabolic activity of fermenting organisms. To overcome the challenge of bioconversion in the presence of inhibitory compounds, especially at high solids loading, a strain of *Saccharomyces cerevisiae *was subjected to evolutionary engineering and adaptation for fermentation of pretreated pine wood (*Pinus taeda*).

**Results:**

An industrial strain of *Saccharomyces*, XR122N, was evolved using pretreated pine; the resulting daughter strain, AJP50, produced ethanol much more rapidly than its parent in fermentations of pretreated pine. Adaptation, by preculturing of the industrial yeast XR122N and the evolved strains in 7% dry weight per volume (w/v) pretreated pine solids prior to inoculation into higher solids concentrations, improved fermentation performance of all strains compared with direct inoculation into high solids. Growth comparisons between XR122N and AJP50 in model hydrolysate media containing inhibitory compounds found in pretreated biomass showed that AJP50 exited lag phase faster under all conditions tested. This was due, in part, to the ability of AJP50 to rapidly convert furfural and hydroxymethylfurfural to their less toxic alcohol derivatives, and to recover from reactive oxygen species damage more quickly than XR122N. Under industrially relevant conditions of 17.5% w/v pretreated pine solids loading, additional evolutionary engineering was required to decrease the pronounced lag phase. Using a combination of adaptation by inoculation first into a solids loading of 7% w/v for 24 hours, followed by a 10% v/v inoculum (approximately equivalent to 1 g/L dry cell weight) into 17.5% w/v solids, the final strain (AJP50) produced ethanol at more than 80% of the maximum theoretical yield after 72 hours of fermentation, and reached more than 90% of the maximum theoretical yield after 120 hours of fermentation.

**Conclusions:**

Our results show that fermentation of pretreated pine containing liquid and solids, including any inhibitory compounds generated during pretreatment, is possible at higher solids loadings than those previously reported in the literature. Using our evolved strain, efficient fermentation with reduced inoculum sizes and shortened process times was possible, thereby improving the overall economic viability of a woody biomass-to-ethanol conversion process.

## Background

Cellulosic ethanol might serve as a sustainable biofuel that could replace gasoline use as a transportation fuel [1,2], and it can be generated from a variety of cellulosic biomass types, such as wood [3]. One challenge that is particularly acute with woody biomass, such as pine, is that the pretreatment process releases a number of compounds that are inhibitory to the growth and/or metabolic activity of the fermenting organism [4]. These chemicals act through a variety of mechanisms to reduce ethanol production efficiency, including inhibition of cell growth, reduction of cell metabolic activity, or inhibition of enzymatic activity. Thus, it is important to use a fermenting organism that is able to tolerate these compounds, especially at the high solids loadings required for industrial fermentations to produce the ethanol concentrations necessary for cost-effective distillation.

Inhibitors found in biomass fermentations are determined by conditions used during pretreatment (temperature, pH, time, and any chemicals used), and act in various ways to inhibit efficient fermentation of sugars to ethanol [5-10]. Inhibitors can be divided into three general categories: aromatic compounds, furan derivatives, and weak aliphatic acids. Aromatic compounds, such as vanillin and 4-hydroxybenzaldehyde, are generated when the lignin in the wood is degraded [11]. Furan derivatives are generated from sugar portions of the feedstock during pretreatment: with furfural (FF) from degradation of pentose sugars, and 5-hydroxymethylfurfural (HMF) from hexose sugars [12]. HMF can be further degraded during pretreatment to produce the weak acids levulinic acid and formic acid. Acetic acid, another weak acid, is formed by hydrolysis of hemicellulose. HMF and FF can decrease ethanol yield and productivity, and slow the organism's growth [6]. FF and HMF act synergistically to decrease ethanol production [7]. The most concentrated weak acids present in pine-wood fermentations are acetic, levulinic, and formic acids, acting to inhibit cellular activity by mechanisms of uncoupling and intracellular anion accumulation [8]. Uncoupling results in a dissipation of the cell's proton gradient; thus hindering its ability to generate ATP [9]. During intracellular anion accumulation, the undissociated form of the acid will diffuse across the plasma membrane, and then dissociate inside the cell, thus decreasing the cytosolic pH [10]. The cell must then correct this pH imbalance. Mechanisms by which aromatics inhibit are not completely elucidated, presumably due to the complex structure of lignin. Proposed mechanisms include a loss of integrity in the cell membrane, and destruction of the electrochemical gradient by transporting protons back into the mitochondria similar to the weak acids [9,13]. Furthermore, it has been shown that FF and aromatic compounds can lead to reactive oxygen species that can randomly oxidize proteins, lipids, and other structures in *Saccharomyces cerevisiae*, and if the damage is too great, the cells will not survive [6,14].

Inhibitory compounds may be removed before fermentation, resulting in increased ethanol production [4,15,16]. Although effective, ameliorating these compounds from fermentations increases overall production costs. The ethanologenic yeast, *S. cerevisiae*, displays relatively robust growth in the presence of inhibitory compounds [17], although the response of individual strains varies widely [18]. Some *Saccharomyces *strains convert HMF to the less toxic 2,5-*bis*-hydroxymethylfuran [19], and the *ADH6 *gene product (alcohol dehydrogenase 6) has been shown to increase the rate at which cells metabolize HMF [20]. *S. cerevisiae *is also able to partially metabolize some of the phenolic compounds, probably via phenylacrylic acid decarboxylase conversion of cinnamic, *p*-coumaric, and ferulic acids to their less toxic vinyl derivatives [21,22]. Furan reductase or laccase have been expressed in yeast [23,24], and these increased fermentation rates. Other efforts to reduce the detrimental effects of inhibitors include optimizing process configurations, such as using fed-batch pulse feeding of hydrolysate instead of immersing the yeast in hydrolysate all at once. *Saccharomyces *strains are able to adapt to some degree if precultured on hydrolysate or via cell recycling [25-27], although the exact mechanisms that result in increased performance are still unknown for many strains.

Previous efforts have described approaches to improve fermentation performance of *S. cerevisiae *strains with respect to inhibitor tolerance. When an industrial strain of *S. cerevisiae *was cultured in increasing concentrations of FF, the time spent in lag phase by the adapted strain was significantly reduced compared with the parental strain [28]. In a later study, this reduction in lag phase was attributed to increased oxireductase activity in the evolved strain [29]. Other researchers have increased xylose utilization in engineered strains through a process called chemostat evolution [30]. In this process, the strain was kept under constant xylose limitation in a chemostat, and the resulting pressure selected for strains that are best able to use xylose as a carbon source. Because of the large natural biodiversity in *S. cerevisiae*, other approaches have focused on the isolation from distilleries of natural strains with the desired phenotypes [31].

In this paper, we describe the directed evolution and adaptation of an industrial *Saccharomyces *yeast strain, XR122N, currently used in corn-ethanol fermentations for the production of ethanol from pretreated lignocellulose. We selected sulfur dioxide-pretreated pine wood as the substrate, because of the high level of inhibitory compounds found in this feedstock. In order to generate a strain with improved tolerance of inhibitory compounds found in pretreated pine, XR122N was evolved using SO_2_-pretreated pine directly, without separating the liquid from the solids and without ameliorating the toxic compounds, rather than using a single inhibitory compound such as FF for directed evolution. The strain was also subjected to additional evolutionary adaptation at high solids loadings in order to increase ethanol concentrations in the fermentation. Growth and ethanol production of the evolved strain in various combinations of 13 inhibitory compounds found in pretreated pine was also investigated. The final evolved strain, AJP50, possesses greater fermentation capability than its parent in both rich liquid media supplemented with various combinations of inhibitory compounds, and in pretreated pine fermentations at high solids loadings.

## Results and Discussion

### Pine fermentations with the industrial yeast strain XR122N

Fermentations using pine pretreated with SO_2 _steam explosion (without washing or inhibitor removal) as the substrate at dry weight solids loadings of 5, 10, and 12% w/v were conducted using the industrial *S. cerevisiae *yeast strain XR122N (North American Bioproducts Corporation, Duluth, GA, USA). Compositional analysis of the pine before and after pretreatment is provided in Table 1 and the list of 13 inhibitory compounds and their concentrations in the pretreated pine sample used for fermentations are listed in Table 2. Freeze-dried XR122N was inoculated at an initial concentration of 4 g/L dry cell weight (dcw), similar to its use in corn-ethanol fermentations, and enzymes for biomass saccharification were added simultaneously with the inoculum (15 filter paper units (FPU) cellulase and 60 cellobiase units (CU) cellobiase per gram dry weight (gdw) of pretreated pine).

**Table 1:** Compositional analysis of pine subjected to sulfur dioxide steam explosion^a^

Sample	Glucan	Xylan	Mannan	ASL^b^	AIL^c^	Sum
Untreated Pine ^d^	42.9	6.0	12.9	0.5	33.2	99.1^d^
3.3% SO_2 _213°C^e^	53.0	1.2	0.4	0.4	44.0	99.0

^a^Shown as percentage of each component on a dry weight basis

^b^Acid-soluble lignin.

^c^Acid-insoluble lignin.

^d^Before pretreatment, the pine wood also contained 2.5%galactan and 1.1% arabinan, which were not detected pine-wood sample after pretreatment. Sum includes the galactan and arabinan fractions.

^e^Reaction time of 5 minutes in SO_2_; percentage and temperature indicated.

**Table 2:** Concentrations (g/L) of each inhibitory compound studied, divided into classes

Furans			Aromatics			Acids		
	PH^a ^	Model^b^		PH^a ^	Model^b^		PH^a ^	Model^b^
HMF^c^	2.153	2.000	3,4-DHBA^d^	0.003	0.003	Formic acid	0.425	0.400
Furfural	1.180	1.000	3-HBA^e^	0.005	0.005	Lactic acid	0.100	0.100
Furoic acid	0.018	0.020	Vanillic acid	0.050	0.050	Acetic acid	2.153	2.000
			Vanillin	0.022	0.020	Succinic acid	0.028	0.030
			Benzoic acid	0.015	0.015	Levulinic acid	0.410	0.400

^a^Concentration of compound measured in pretreated pine hydrolysate (PH) used in 12% w/v solids fermentations.

^b^Concentration of compound in model inhibitor medium.

^c^Hydroxymethylfurfural.

^d^Dihydroxybenzaldehyde.

^e^Hydroxybenzaldehyde.

**Figure 1:**
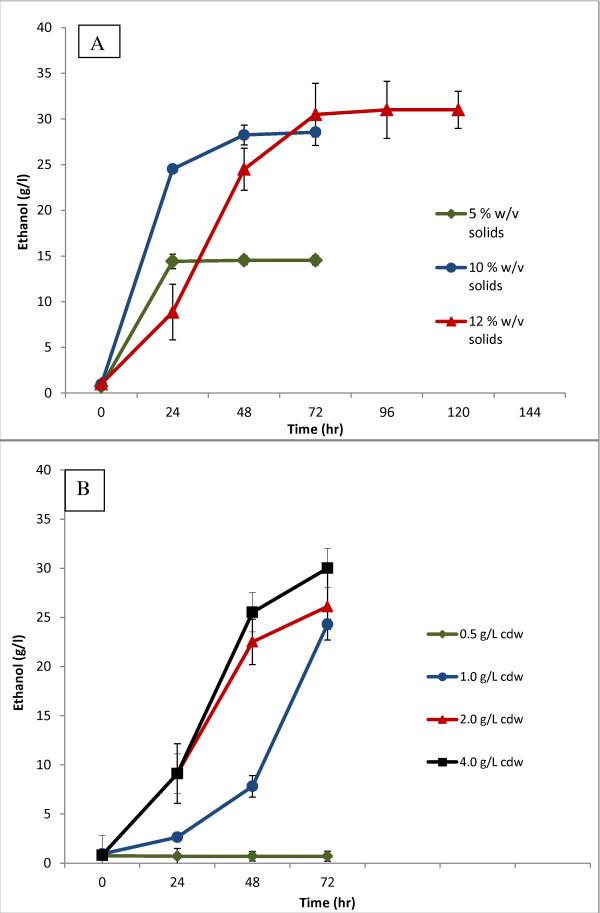
**Effect of solids loading on *Saccharomyces cerevisiae *strain XR122N**. (A) Freeze-dried XR122N was inoculated at an initial concentration of 4 g/L cell dry weight (dcw) into small-scale bioreactors containing pretreated pine at a solids loading of 5% (green diamonds), 10% (blue circles), and 12% (red triangles) w/v. Cellulases and cellobiase were added simultaneously with the inoculum (15 FPU cellulase, and 60 CU cellobiase per gdw of pretreated pine). (B) XR122N was inoculated into 12% w/v solids loading of pretreated pine in a freeze-dried state at an initial concentration of 4 g/L dcw (black squares), 2.0 g/L dcw (red diamonds), 1.0 g/L dcw (blue circles), or 0.5 g/L dcw (green diamonds). Fermentations were maintained at 35°C, pH 5.0, and performed in triplicate. Error bars represent one standard deviation from the mean. FPU, filter paper units; dcw, cell dry weight; gdw, gram dry weight; CU, cellobiase units.

Simultaneous saccharification and fermentation (SSF) was desired for fermentations, because the added enzymes release monomeric forms of carbohydrates from the solids content of pretreated pine, and the fermenting yeast consumes the sugars as soon as they are released, thus minimizing end-product inhibition [32,33]. The optimal conditions for the fungal enzyme preparations used in these experiments are a pH of 4.5 and a temperature of 45°C, conditions too harsh for the fermenting yeast. Thus, to optimize enzyme activity during SSF experiments while minimizing the effects on the yeast, the pH was held at 5.0, just slightly above the enzyme optimum pH, and the temperature for fermentation decreased from 45°C to between 35 and 37°C. Attempts to increase the fermentation temperature above 37°C dramatically reduced ethanol production (data not shown). Ethanol production from the different biomass concentrations is presented in Figure 1A.

The effect of inoculum size on pretreated pine fermentations at a 12% w/v solids loading is presented in Figure 1B. Initial attempts at inoculation of pretreated pine solids at or above 5% w/v using a low inoculum level equal to 0.2 g/L dcw resulted in cell death of XR122N (absence of growth on solid or liquid medium) and no ethanol was detected in these cultures. An inoculum size of 0.5 g/L produced ethanol in pretreated pine fermentations at a 10% w/v solids concentration (data not shown), but at a 12% w/v solids concentration no ethanol production was detected (Figure 1B). Increasing the inoculum level to 1 g/L dcw in 12% w/v solids fermentations resulted in ethanol production, albeit with a pronounced lag phase of 72 hours. An inoculum of 2 g/L dcw produced almost as much ethanol as 4 g/L, and was selected as the inoculum size for further studies.

One of the most promising pretreatments for softwoods, including pine, spruce, and Douglas fir, is SO_2 _steam explosion [34], and various combinations of SO_2 _concentration_, _reactor temperature, and time of reaction have been published. Table 3 compares the available data on SO_2 _single-step pretreatments followed by SSF to produce fuel ethanol. Owing to the toxic nature of the pretreated softwood, many of the fermentations were conducted with solids loadings of 5 to 12% w/v. The inoculum level for yeasts was routinely 4 to 5 g/L and enzyme loadings ranged from 0 to 42 FPU cellulase per gdw of cellulose. Many softwood fermentations were conducted using washed solids [35,36], diluted solids with filtration [37], or lower solids loading of 5 to 8% w/v dry matter [25,35]. Hoyer and colleagues [38] obtained excellent results (94.7% of maximum theoretical yield based on glucose and mannose in the pretreated material) during fermentations with 10% w/v solids content. However, when using the same material at 12% w/v dry-matter solids loading, the maximum theoretical ethanol yield decreased to only 37%. All of these previous studies highlight the difficulties involved in fermenting pretreated softwood. Similarly, in the present study, we saw a decrease in the maximum theoretical yield (from 98% to 76%) when the dry-matter solids loading was increased from 10% to 12% w/v.

**Table 3:** Comparison of simultaneous saccharification and fermentation methods using SO_2 _pretreatment of softwoods with *Saccharomyces cerevisiae *strains

Yeast strain^a^	Wood type	Pretreatment^b^			Solids, % dry weight/volume	Inoc, g/L	Cellulase, FPU/gram dry weight	Max EtOH, g/L	Time to maximum EtOH production, hours	% TM	Reference
		**SO_2 _conc**.	Reaction temp, °C	Duration, min							
Tembec 1	Lodgepole pine	4.0	200	5	5% (washed)	5	40 FPU/g cellulase, 20 CU/g cellobiase	17.0	24	68^c^	Ewanick *et al. *[36] (note: 6 hours enzyme preincubation)
Tembec 1	Douglas fir	4.5	195	4.5^d^	40 mL, WSF	5	No enzymes added	13.8	24	87^e^	Keating *et al. *[37]
Y-1528	Douglas fir							14.7	24	92^f^	
Baker's yeast	Spruce	2.5	215	5	8%	5	32 FPU/g cellulase, 28 IU/g cellobiase	Not stated	72	60^f^	Alkasrawi *et al. *[25]
Baker's yeast^g^									72	92^f^	
TMB 3000									48	89^f^	
Baker's yeast	Spruce	3.0	215	5	5%	5	15 FPU/g cellulose, 23 CU/g cellobiase	Not stated	24	49^f^	Söderström *et al. *[35]
Baker's yeast	Spruce	3.0	210	5	12% WIS	5	15 FPU/g cellulose, 23 CU/g cellobiase	20.0 (graph)	72	37^f^	Hoyer *et al. *[38]
XR122N	Pine	3.3	215	5	10%	4	15 FPU/g cellulose, 60 CU cellobiase	28.7	48	98^c^	This study
XR122N	Pine	3.3	215	5	12%	4	15 FPU/g cellulose, 60 CU cellobiase	23.6	48	76^c^	This study

Abbreviations: FPU, filter paper unit; Inoc, inoculation; CU, cellobiase unit; temp, temperature; WIS, water-insoluble solids; WSF, water-soluble fraction.

^a^*Saccharomyces*.

^b^All biomass was pretreated with SO2 in a single step and was not delignified before pretreatment or fermentation.

^c^Theoretical yield based on cellulose and hemicellulose content derived from gravimetric and analytical analyses of pretreated material.

^d^Steam explosion output was diluted to 15% w/w and filtered, and the pH altered to 6.0. Suspended solids not filtered out.

^e^Theoretical yield was based on the content of glucose and mannose in the pretreated material.

^f^Theoretical yield was based on the content of glucose and mannose in the liquid and of glucan in the solid material.

^g^In hydrolysate.

### Evolution of XR122N for fermentation at high solids loading

To reach the ethanol concentrations necessary for cost-efficient distillation, the solids loading must be 15 to 20% w/v [39]. However, as the biomass content increases in the fermentation, the concentration of inhibitory compounds also increases. Previous studies with *Saccharomyces *spp. illustrated that some strains are able to adapt to varying degrees by preculturing on hydrolysate or via cell recycle [25-27]; the exact mechanisms for increased performance are still unknown for many of these strains. Using FF alone for adaptation experiments results in different phenotypes, depending upon the method used for selection. In previous work, increased rates of FF reduction were seen in selection regimens in which FF was added during logarithmic growth [40]. By contrast, challenging cells at a low inoculum size to relatively high concentrations of FF did not change the FF reduction rates, but significantly reduced the lag phases and allowed growth in glucose minimal medium containing 40% v/v of spruce acid hydrolysate sample, a medium that killed the parental strain [28].

**Figure 2:**
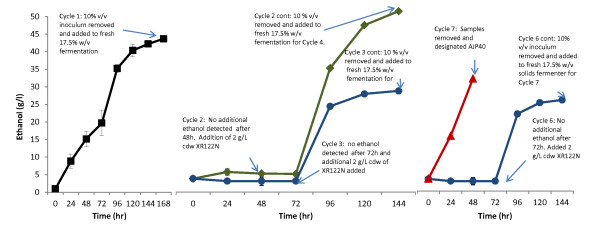
**Evolution and adaptation of XR122N to high solids loading of pretreated pine to produce AJP40**. XR122N was inoculated into a 17.5% w/v solids fermentation, and allowed to ferment for 168 hours. 10% v/v aliquots were removed as indicated in the text, and used to inoculate 17.5% w/v solids fermentations. Ethanol was measured every 24 hours, and an additional 2 g/L cdw of XR122N added as indicated. After six full cycles the pattern changed, and ethanol was produced after 24 and 48 hours of fermentation without the addition of more XR122N cells. Cycles 4 and 5 had identical performance to cycles 2 and 3, and are omitted for clarity.

In the present study, inoculation of high solids loading (>10% w/v solids) using a low inoculum level of XR122N provided multiple stressors (increase in particulate content and inhibitory compounds), and selection was targeted at yeast survival and ethanol production. Directed evolution experiments were started at a concentration of 2 g/L dcw inoculum of XR122N added to pretreated pine fermentations at a 17.5% w/v solids loading as described in Methods (Figure 2). Fermentation was stopped after 168 hours, and aliquots equal to 10% v/v were transferred to fresh 17.5% w/v fermentations. When ethanol was not detected after 96 hours and aliquots from the fermenters did not exhibit growth in yeast-peptone-dextrose (YPD) media, 2 g/L dcw of XR122N cells were added to the fermentation vessels. Ethanol production was detected after another 24 hours of fermentation in one vessel, and continued to increase for an additional 48 hours. A 10% v/v inoculum (approximately 1 g/L dcw) was removed from the fermentation vessel where ethanol production was detected, and used to inoculate a third fermentation vessel containing 17.5% w/v pretreated pine and enzymes. No additional ethanol was produced after 96 hours, even though aliquots of the cells grew in liquid media. Another 2 g/L dcw of XR122N was then added to the fermentation. This process of inoculating a 17.5% w/v solids fermentation with a 10% v/v inoculum from a previous fermentation, monitoring ethanol production for 96 hours without observing an increase in ethanol content, and adding 2 g/L dcw of XR122N, was repeated for a total of six full cycles. During the seventh cycle, ethanol production increased by 24 hours, and continued to increase at 48 hours. At 48 hours of fermentation, a 10% v/v inoculum was transferred to a fresh 17.5% w/v solids fermentation and ethanol production monitored. Samples from this fermentation (removed after 48 hours) were frozen in glycerol at -80°C, and designated AJP40 (Figure 2). A similar set of fermentations using 20% w/v solids failed to produce high concentrations of ethanol, even after the addition of 2 g/L dcw of XR122N (data not shown).

**Figure 3:**
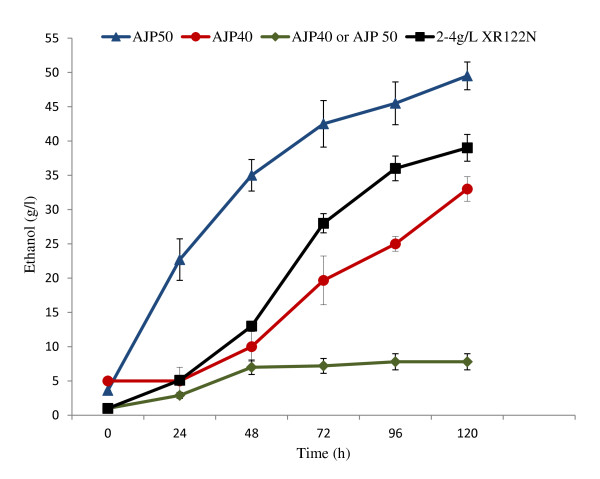
**Comparison of AJP50 and AJP40 in fermentations with 17.5% w/v pretreated pine solids loading**. Direct inoculation of AJP40 or AJP50 from freezer stocks (approximately 0.2 g/L dcw; green diamonds) was carried out. A short adaptation protocol of inoculation (0.2 g/L dcw) into 7% w/v solids for 24 hours was used, and a 10% v/v aliquot (approximately 1 g/L dcw) from this fermentation was used to inoculate 17.5% w/v solids using either AJP40 (red circles) or AJP50 (blue triangles). The curve of XR122N (black squares) at 2 to 4 dcw/L is provided for comparison.

AJP40 was subjected to additional transfers in 17.5% w/v solids loading of pretreated pine. Inoculation of AJP40 into 17.5% w/v solids directly produced little ethanol (Figure 3); however, if 10% v/v aliquots from this unproductive fermentation were inoculated into less concentrated solids, ethanol was produced (data not shown). Inoculation of AJP40 glycerol stocks (approximately 0.2 g/L dcw) into a 7% w/v solids fermentation resulted in the maximum theoretical yield of ethanol production after 24 hours of fermentation, and a 10% v/v aliquot was used to inoculate a 17.5% w/v solids fermentation. Ethanol production was seen at 48 hours, and when a 10% v/v inoculum of this fermentation was transferred into a fresh 17.5% w/v solids fermentation, ethanol was detected after 24 hours. Additional transfers into 17.5% w/v solids were made as described in Methods, for a total of 50 transfers.

The resulting strain exhibiting the phenotype of increased ethanol production and decreased lag time in high solids fermentations was designated AJP50, and used for subsequent studies. Inoculation of 17.5% w/v solids fermentations with AJP50 taken directly from revived freezer stocks (0.2 g/L dcw) did not produce ethanol levels of above 10 g/L. However, inoculating AJP50 (0.2 g/L dcw) into a fermentation with reduced (7% w/v) solids loading for a short adaptation period (24 hours), followed by removal of a 10% v/v inoculum (approximately 1 g/L dcw) into 17.5% w/v solids fermentations, improved ethanol production significantly (Figure 3). With this short adaptation period, the evolved strain, AJP50, had a reduced lag time and produced over 80% of the maximum theoretical yield in 72 hours of fermentation and over 90% of the maximum theoretical yield of ethanol in 120 hours.

### Growth and ethanol production in the presence of inhibitors

**Figure 4:**
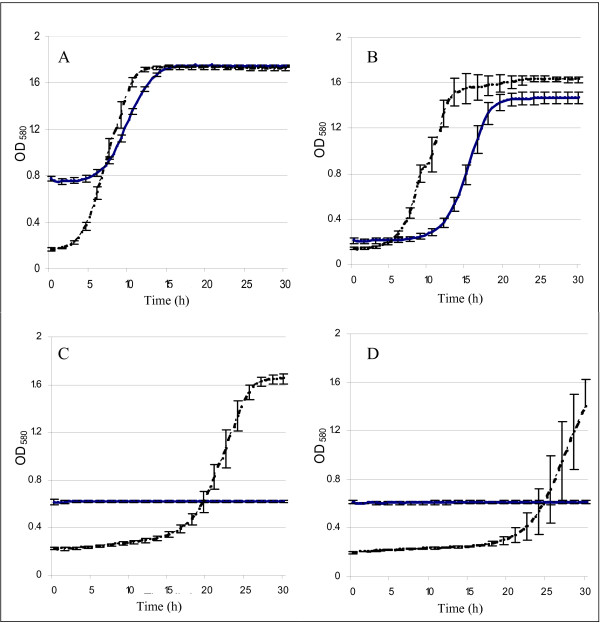
**Growth of XR122N (solid blue) and AJP50 (dashed black) in the presence of various mixtures of inhibitory compounds found in biomass fermentations**. Growth in **(A) **the aromatic mixture; **(B) **the acid mixture; **(C) **a combination of hydroxymethylfurfural (HMF), furfural (FF), and acetic acid; and **(D) **the furan mixture. All compounds and their concentration in the media are shown in Table 2. The inhibitory compounds were dissolved in tryptic soy broth with 2% w/v glucose. Error bars represent one standard deviation from the mean. XR122N required a higher volume of revived culture to obtain a cell concentration equivalent to 4.0 × 10^5 ^cells/ml; this increased the wood particulate matter in the culture and led to the higher initial optical density seen in some XR122N cultures.

AJP50 appeared to have acquired the ability to grow and ferment high concentrations of solids, but with increasing solids concentrations, there were increased amounts of inhibitory compounds present as well. To determine whether AJP50 had an advantage over the parental strain in the presence of inhibitory compounds, we compared the growth profiles of both strains in different combinations of inhibitory compounds typically found in biomass fermentations (Table 2). Growth of both AJP50 and XR122N was not inhibited by the aromatic mixture and was very weakly inhibited by the acid mixture under concentrations tested (Figure 4A,B). Low concentrations of weak acids have been shown to stimulate ethanol production in *S. cerevisiae*, but high concentrations were inhibitory to the activity of the organism in previous studies [5,15].

The inhibitory factors present in the largest concentrations in biomass fermentations are HMF, FF, and acetic acid, thus both strains were also grown in the presence of a mixture of these; the parental strain was strongly inhibited while the evolved strain showed an increase in lag phase. (Figure 4C). Growth of both strains was strongly inhibited by the mixture of the furan compounds HMF, FF, and furoic acid (Figure 4D). With this combination, no growth of XR122N was seen over 30 hours. Growth of AJP50 had a longer lag phase than in the other conditions; however, the furan-inhibited AJP50 cultures did eventually reach the same final optical density (OD) as the uninhibited cultures.

The effects of FF and HMF on certain strains of *S. cerevisiae *have been described previously by a number of groups [12,19,41]. FF completely inhibited the growth of yeast strains at a concentration of 5.76 mg/ml. and partially inhibited growth at a concentration of 2.88 mg/ml during an incubation period of 125 hours. HMF completely inhibited one strain, and partially inhibited another at 7.6 mg/ml; various degrees of partial inhibition were seen at a concentration of 3.8 mg/ml. These concentrations are higher than those previously reported for pine-wood biomass fermentations, however, the amounts of inhibitory compounds might increase with increased severity of the pretreatment, and with increased concentrations of biomass at high solids loadings.

**Figure 5:**
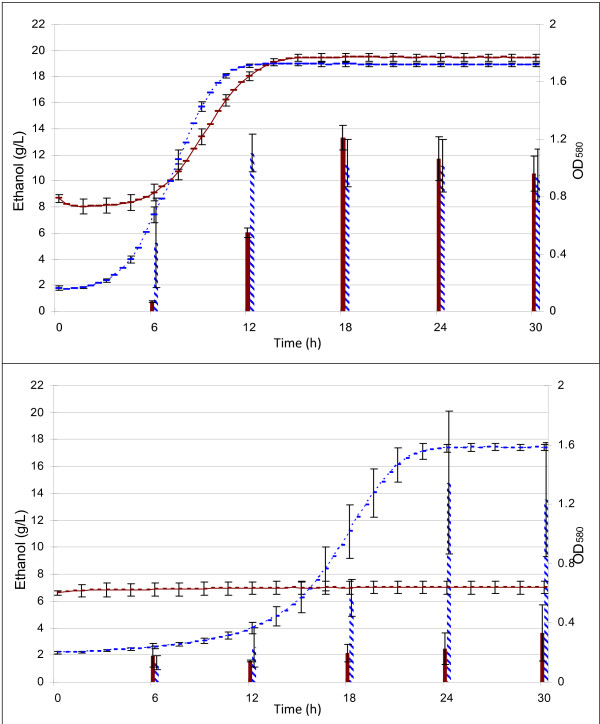
**Growth and ethanol production of both strains in the presence or absence of the selected inhibitory compounds present in pine-wood biomass fermentations**. Lines represent cell culture density measured at an optical density of 580 nm (secondary *y *axis). Bars represent ethanol concentration in g/L (primary *y *axis). The red, solid and blue, dashed lines and bars show data from XR122N and AJP50 at 4.0 × 10^5 ^cells/ml initial cell density. Growth and ethanol production in the (upper panel) absence of inhibitors and (lower panel) in the presence of all 13 inhibitory compounds are shown.

To further study inhibition of the strains by these compounds, ethanol production of both strains was compared in the presence of 13 inhibitory compounds (Table 2) and in the absence of any inhibitors. Growth data were compared with ethanol data for both strains (Figure 5). Even though XR122N failed to grow in the presence of all 13 compounds, it still produced a small amount of ethanol after 30 hours. By contrast, AJP50 produced the theoretical maximum concentration of ethanol at 18 hours. Interestingly, in the absence of inhibitory compounds, AJP50 was able to produce ethanol after 6 hours and reached a maximum at 12 hours, whereas XR122N did not produce ethanol until 12 hours, and took 18 hours to reach maximum.

### Conversion of furfural and hydroxymethylfurfural to alcohol derivatives

**Figure 6:**
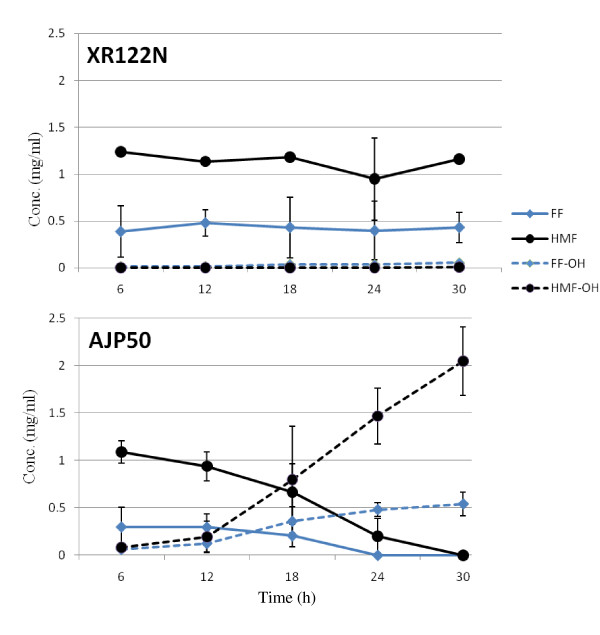
**Conversion of furfural (FF) and hydroxymethylfurfural (HMF) to their less toxic alcohol derivatives**. XR122N and AJP50 were compared for their ability to convert FF and HMF to their less toxic alcohol derivatives FF alcohol (FF-OH) and HMF alcohol (HMF-OH). The concentration of each compound is presented for each organism during a 30-hour fermentation in tryptic soy broth with 2% w/v glucose.

A similar approach to the one used to generate AJP50 was used by Martín and colleagues to adapt *S. cerevisiae *to the inhibitory compounds in sugar-cane bagasse [27]; their study used media with known concentrations of inhibitors added, whereas in the present study, we used pretreated biomass as the media for adaptation and further directed evolution. In the study by Martín *et al*., the advantage of the evolved strain was attributed to its ability to more rapidly detoxify FF and HMF [27]. Heer and Sauer were able to evolve another *S. cerevisiae *strain to FF alone, and this evolved strain had a marked decrease in lag phase, later attributed to increased action of certain oxireductases [28,29]. Although we used pretreated biomass for the evolutionary adaptation instead of FF or HMF directly, the resulting strain AJP50 is also able to rapidly detoxify FF and HMF by converting them to their less toxic alcohol derivatives (Figure 6).

### Stability of the AJP50 inhibitor-resistant phenotype on rich media

To determine if AJP50 would retain its phenotype during routine culturing, the strain was cultured on YPD media without inhibitory compounds. The ability of the resulting culture to grow in inhibitory media was then assessed. After culturing on rich solid and liquid media, AJP50 maintained resistance to the effects of inhibitors found in lignocellulosic biomass fermentations (Table 4). After 24 hours of growth, 14 of 100 cultures had an optical density of greater than 1.5, and 40 cultures had an OD of between 1.2 and 1.5. ODs of this level indicate resistance to the inhibitory compounds; XR122N cultures uniformly have ODs of less than 0.3 after 24 hours of growth under these conditions. Only six cultures had optical densities of less than 0.3, indicating that only a few cultures displayed no resistance after culturing on YPD media. After 30 hours of growth, the number of cultures with an OD of greater than 1.5 had increased to 43; at this time point only two cultures possessed ODs of less than 0.3, and 91% of the cultures had ODs greater than 1.2. However, multiple transfers of AJP50 onto YPD media results in a widely variable loss of the inhibitor-resistant phenotype (data not shown), thus for this reason YPD was supplemented with all 13 inhibitors in the following experiments, to act as a selective pressure, causing AJP50 to invariably retain its phenotype during routine culturing and isolation.

**Table 4:** Optical densities at OD_580_ of AJP50 cultures in inhibitory media after growth on rich media

OD_580_	24 hours	30 hours	48 hours
0.0 to < 0.3	6	2	2
0.3 to <0.6	5	1	0
0.6 to < 0.9	13	2	0
0.9 to < 1.2	22	4	6
1.2 to < 1.5	40	48	50
≥1.5	14	43	42

^a^Values are a percentage of the cultures out of 100 replicates

### Analysis of isolated clones and verification of the inhibitor-resistant phenotype

**Figure 7:**
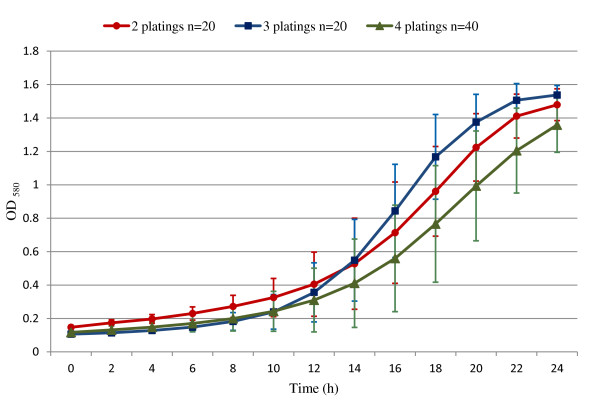
**Verification of phenotype in individually isolated clones from the evolved yeast population**. Glycerol freezer stock samples from AJP50 fermentations plated onto yeast-peptone-dextrose (YPD) agar containing all 13 inhibitory compounds (YPD-AI) yielded isolated colonies. Individual colonies were then subcultured on additional YPD-AI once (two platings), twice (three platings), or thrice (four platings), as described in Methods. Isolated colonies were grown in YPD broth containing all 13 inhibitory compounds (YPD-BI). Growth curves for each series of platings was plotted as a single line with error bars representing one standard deviation from the mean (two platings (red), n = 20; three platings (blue), n = 20; four platings (green), n = 40). XR122N did not grow in YPD-BI or YPD-AI, and is not represented on the graph.

To verify the phenotype of individually isolated clones from the evolved yeast population, samples of AJP50 fermentations with 7% w/v solids were grown in YPD broth containing all 13 inhibitory compounds (YPD broth + inhibitors; YPD-BI) and then plated onto YPD agar containing all 13 inhibitory compounds (YPD agar + inhibitors; YPD-AI) to obtain isolated colonies. Individual colonies from these plates were subcultured onto a second YPD-AI plate for isolation. Individual colonies from the second plate were either inoculated into YPD-BI for growth-curve experiments, or plated again to produce isolated colonies. The growth curves of individual colonies plated for isolation on a series of two, three, or four plates, prior to inoculation for growth-curve measurements, were plotted (Figure 7). The results were similar for replicates within the same plating series, and all individual growth curves for each type of plating regimen are plotted as one line, with error bars depicting one standard deviation from the mean. ODs of greater than or equal to 1.2 after 24 hours of growth in YPD-BI indicates resistance to the inhibitory compounds. XR122N did not grow in YPD-AI or YPD-BI, and is omitted from the graph (Figure 7).

### Reactive oxygen species in AJP50 and XR122N cultures incubated with inhibitory compounds

**Figure 8:**
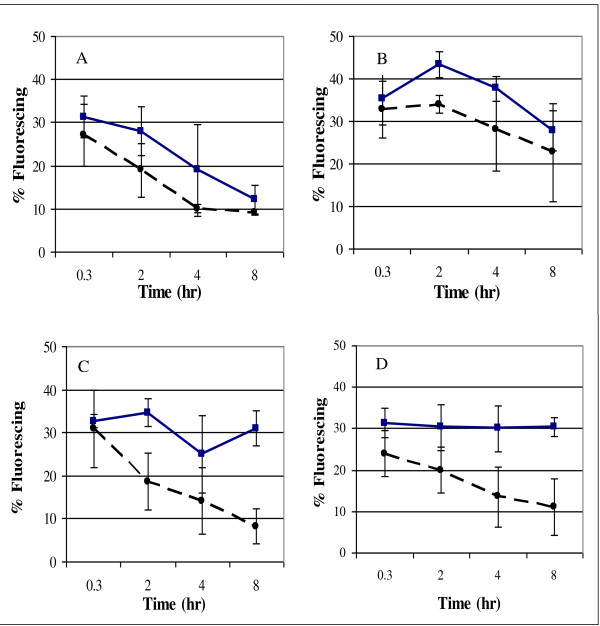
**Levels of reactive oxygen species in XR122N and AJP50 cultures grown in media containing biomass inhibitors**. XR122N is represented by solid blue lines and squares, AJP50 by dashed black lines and circles. At each time point, at least 100 cells were surveyed and, the percent of cells fluorescing was recorded. Data from **(A) **uninhibited control; and cultures grown in media containing **(B) **5 mmol/l H_2_O_2_, **(C) **hydroxymethylfurfural (HMF), furfural (FF), and acetic acid, **(D) **and all 13 inhibitors listed in Table 1. Error bars represent one standard deviation from the mean.

Both XR122N and AJP50 underwent considerable damage from reactive oxygen species (ROS) when revived in media from glycerol stocks at -80°C. AJP50 was able to recover from this damage more rapidly than XR122N in the presence of inhibitory compounds found in biomass fermentations (Figure 8). XR122N was able to reduce its level of ROS in the absence of inhibitory compounds, but was only able to slightly alleviate the ROS damage in the presence of inhibitors. In the presence of all 13 inhibitors and the mixture of HMF, FF, and acetic acid, AJP50 experienced similar levels of ROS to those seen in the absence of these compounds. XR122N and AJP50 experienced similar levels of recovery from ROS in the uninhibited and H_2_O_2_-supplemented controls, indicating that the faster reduction of ROS by AJP50 in the inhibitory media is related to the presence of the inhibitory compounds.

## Methods

### Pretreatment of pine-wood biomass

Loblolly pine from Georgia, USA, was debarked and chipped to a particle size of 10 mm or smaller. The chips were pretreated with gaseous sulfur dioxide [42,43], and subjected to steam explosion in the Process Development Unit (PDU) at the Chemical Engineering Department, Lund University, Sweden, or in a similar PDU located at the Georgia Institute of Technology under the direction of C2Biofuels (Atlanta, GA). A known weight of chips was pretreated with 3.3% SO_2 _(w/w moisture content of chips) and held at 215°C for 5 minutes in the PDU in a single-step process. The resulting material consisted of a mixture of liquids and solids. These phases were not separated, pressed, drained, or washed to remove potentially inhibitory compounds. Materials were stored at 4°C until use.

### Compositional analysis

Determination of the structural carbohydrates, lignin, sugars, byproducts, and degradation products were determined using National Renewable Energy Laboratory (NREL) Biomass Program methods [44]. Moisture content was determined using a moisture analyzer (IR-35; Denver Instrument, Denver, CO, USA), and all fermentation loadings were determined on a dry-matter basis, referred to as the percentage w/v of pretreated pine. Samples were analyzed by HPLC (Shimadzu, Kyoto, Japan) with refractive index detection, essentially previously as described [45]. Briefly, monomeric sugars were separated using an Aminex HPX-87P column (Bio-Rad, Hercules, CA, USA), at 80°C, with a flow rate of 0.5 ml/min and a water mobile phase. Samples were filtered (0.22 μm) before analysis. The percentage of fermentable carbohydrates was defined as the sum of the estimated cellulose and hemicellulose values. Although yeasts used in these studies do not ferment pentose sugars, the low xylose content of the pretreated pine was included in the theoretical yield calculation and in the 'fermentable carbohydrate' total. The percentage of maximum theoretical yield was calculated by the following formula:

total fermentable carbohydrate×dry weight of pine×0.53 (molecular ratio of ethanol/polymer carbohydrate)×0.9(conversion efficiency of 6C sugars).

### Pretreated pine fermentations with XR122N

Fermentations were performed in small-scale bioreactors with a working volume of 200 mL using pretreated pine-wood biomass as feedstock essentially as described previously [45]. The percentage moisture was determined using the IR-35 moisture analyzer as before and samples containing 5, 10, and 12% w/v dry solids were weighed, added to a 500 ml flask, and autoclaved at 121°C for 20 minutes to ensure sterility (although this could be considered an additional pretreatment). Upon cooling, double-strength tryptic soy broth (TSB, containing 15 g pancreatic digest of casein, 5 g papaic digest of soybean meal, and 5 g NaCl per liter; Difco, Detroit, MI, USA), and sterile water were added, and the pH adjusted to 5.0 with 2 mol/L KOH. The *S. cerevisiae *strain XR122N (North American Bioproducts Corporation, Duluth, GA, USA) was inoculated in a freeze-dried state at an initial concentration of 4 g/L dcw similar to its use in corn-ethanol fermentations. Cellulases and cellobiase (Novozymes Inc., Franklinton, NC, USA) were added simultaneously with the inoculum at concentrations of 15 FPU and 60 CU per gdw of pretreated pine, respectively. Fermentations were maintained at 35°C and pH 5.0, sampled every 24 hours, and ethanol concentration estimated using gas chromatography as previously described [46]. All fermentations were performed in triplicate, and error bars represent one standard deviation from the mean. Inoculation of pretreated pine at 10, and 12% w/v solids loading was performed using 0.2, 0.5, 1, 2, and 4 g/L dcw.

### Evolutionary adaptation of XR122N

A 2 g/L dcw inoculum of XR122N was added to pretreated pine fermentations at a 17.5% w/v solids loading for simultaneous saccharification and fermentation at 37°C and pH 5.0. The fermentation was allowed to proceed for 168 hours, and aliquots equal to 10% v/v were transferred to fresh fermentations containing 17.5% w/v solids, enzymes, and TSB, as described previously. Measurements of cell biomass using optical-density readings or dcw were not possible, because of the particulate matter present from the pretreated biomass, therefore cultures were monitored for ethanol production every 24 hours. Cultures were plated during transfer to the fresh 17.5% w/v solids fermentation, and were approximately equivalent to 1 g/L dcw. After no ethanol was detected at 96 hours, an additional 2 g/L dcw of XR122N cells were added to the fermentation vessels. Ethanol production was measured every 24 hours, and ethanol concentrations in one of the fermentation vessels continued to increase for an additional total of 72 hours. A 10% v/v inoculum was removed from the fermentation vessel in which ethanol production was detected, and used to inoculate a third fermentation vessel containing 17.5% w/v pretreated pine and enzymes. Ethanol production was measured every 24 hours; no additional ethanol was produced after 96 hours of fermentation. Again, another 2 g/L dcw of XR122N was added to the fermentation at this point. This process of inoculating a 17.5% w/v solids fermentation with a 10% v/v inoculum from a previous fermentation, monitoring ethanol production for 96 hours without observing an increase in ethanol content, and adding 2 g/L dcw of XR122N was repeated for a total of six full cycles. During the seventh cycle, measurement at 24 hours showed the ethanol production had increased, and it continued to increase up to 48 hours. At 48 hours of fermentation, a 10% v/v inoculum was transferred to a fresh 17.5% w/v solids fermentation, and the ethanol production monitored. Samples from this fermentation were frozen in glycerol at -80°C, and designated AJP40. A similar set of fermentations using 20% w/v solids failed to produce high concentrations of ethanol, even after the addition of 2 g/L dcw of XR122N.

### AJP40

Glycerol stocks of AJP40 were subjected to additional transfers. First, AJP40 (approximately 0.2 g/L dcw) was inoculated into fermentations containing 17.5% w/v solids loading of pretreated pine; these produced little ethanol. Inoculation of the same amount of AJP40 into a 7% w/v solids fermentation resulted in maximum ethanol production after 24 hours of fermentation, and a 10% v/v aliquot of this fermentation was used to inoculate a 17.5% w/v solids fermentation. Ethanol production was seen at 48 hours, and upon transfer of a 10% v/v inoculum into another 17.5% w/v solids fermentation, ethanol was detected after 24 hours. Additional transfers into 17.5% w/v solids were made by removing a 10% v/v inoculum from a 17.5% solids fermentation that was producing ethanol after 48 hours, and placing it into a new flask containing 17.5% w/v solids and enzymes for saccharification. Transfers were made every 48 hours for a total of 50 transfers. Aliquots from the final (50th) fermentation were frozen in glycerol stocks and designated AJP50.

### Growth in combinations of inhibitory compounds

Stock solutions of each inhibitor were prepared fresh on the day they were to be used. Typical compounds found in pretreated pine wood were grouped by inhibitor class, and were examined in various mixtures. These inhibitory compounds comprised weak acids (acetic, formic, levulinic, lactic, and succinic acids), aromatics (3,4-dihydroxybenzaldehyde, 4-hydroxybenzaldehyde, vanillic acid, vanillin, and benzoic acid), and furans (FF, HMF, and 2-furoic acid). The effects of all 13 compounds were also examined simultaneously, and a mixture of HMF, FF, and acetic acid was also evaluated. The concentrations of each compound were similar to those seen in pretreated pine-wood fermentations (Table 2).

Freezer stocks were created from 7% w/v pretreated pine-wood fermentations for both AJP50 and XR122N. Freezer stocks were revived briefly (<10 minutes) in 9 ml TSB, and microscopic cell counts performed with a hemocytometer were used to standardize the initial inoculum concentration to 4.0 × 10^5 ^cells/ml in each well, which contained 20 g/L glucose and TSB. The starting OD for XR122N appeared to be higher than that of AJP50 because the presence of more particulate matter in the original inoculum from the freezer stocks, thus a larger volume of material was required to obtain an initial cell concentration of 4.0 × 10^5 ^cells/ml for XR122N. The initial pH of each well was 5.0, and temperature was maintained at 37°C in a growth curves machine (Bioscreen C; Oy Growth Curves Ab Ltd. Helsinki, Finland) without shaking. OD of the wells was recorded every 30 minutes at 580nm. Each well was replicated on the plate five times, and used to calculate the mean and standard deviation.

### Ethanol production in model media containing various combinations of inhibitory compounds and glucose as the carbon source

Ethanol production was measured by inoculating wells of a plate with the inhibitor stock to be studied and the culture of interest as described above. Ethanol samples were taken in triplicate at each time point. Ethanol was sampled every 6 hours by removing the plate and removing full 300 μl volume of the appropriate wells by pipette into separate 0.22 μm centrifuge filtration tubes. These were then separated by centrifugation at 10,956 × g for 1 minute at room temperature before being frozen at -20°C until further analysis. Ethanol concentration in the samples was determined using gas chromatography as previously described [46].

### Conversion of furfural and hydroxymethylfurfural to alcohol derivatives

These samples were also evaluated for the conversion of FF and HMF at 6-hour intervals in fermentations described above. FF, HMF, FF alcohol, and HMF alcohol concentrations were determined using HPLC as described previously [34].

### Examination of the inhibitor-resistant phenotype of AJP50

AJP50 was cultured overnight on YPD agar at 37°C and a single colony was used to inoculate a 50-ml flask of YPD broth. The inoculated flask was incubated overnight at 37°C with shaking. The overnight YPD broth culture was examined (Bioscreen) in the presence of all 13 inhibitors as before. All 100 wells of the plate were identical in media composition and initial inoculum level; the OD of the wells was determined at 24, 30, and 48 hours after inoculation to determine how well AJP50 retained its resistance to the inhibitors after culture on rich media lacking any inhibitory compounds. 

### Analysis of isolated clones and verification of inhibitor-resistant phenotype

AJP50 glycerol stocks from the directed evolution were used to inoculate 7% w/v pretreated pine solids fermentations, and incubated at 37°C for 24 hours with shaking. Samples from the 7% w/v solids fermentation were removed, and frozen as 40% w/v glycerol stock cultures. Aliquots of these glycerol stocks were revived in YPD-BI, and incubated for 24 hours at 37°C with shaking. Isolated colonies were obtained by plating onto YPD-AI, and incubated at 37°C. Colonies took an average of 7 days to develop on the YPD-AI plates. Individual colonies from these plates were subcultured onto a second YPD-AI plate, and incubated at 37°C for approximately 7 days. Isolated colonies from this second plate were used to inoculate YPD-BI, and incubated for 24 hours at 37°C with shaking. Aliquots from this broth were used to inoculate wells in plates used for growth curve experiments in the Bioscreen apparatus as described previously, to ascertain if the inhibitor-resistant phenotype was being maintained during isolation and culturing.

A second round of experiments involved selection of isolated colonies from the second YPD-AI plate described above, and subculture onto a third YPD-AI plate. Isolated colonies from the third plate were then inoculated into YPD-BI, and used for growth-curve experiments.

A third set of experiments involved selection of isolated colonies from the third YPD-AI plate, and subculture for isolated colonies onto a fourth YPD agar plate. Isolated colonies were inoculated into YPD-BI, and screened for growth as described previously.

### Comparison of the effect of reactive oxygen species on XR122N & AJP50

The effect of ROS on XR122N and AJP50 was measured using 2' 7'-dichlorofluorescein diacetate (DCF; Sigma-Aldrich Corp., St. Louis, MO, USA), which fluoresces in the presence of ROS, as described previously [14,47]. XR122N and AJP50 were inoculated at 4.0 × 10^5 ^cells/ml from freezer stocks into 50 ml YPD media containing either: all 13 inhibitors; HMF, FF, and acetic acid; 5 mM H_2_O_2_; or no inhibitors. Cultures were maintained at 37°C with shaking, and samples taken at the indicated time points. Samples were examined for fluorescence using a reflected fluorescence microscope (BX61; Olympus Corp., Tokyo, Japan) with a fluorescein isothiocyanate filter. For each time point, at least 100 cells were examined, and the percentage of cells exhibiting fluorescence determined; this reflects the portion of the cell population experiencing ROS damage.

## Conclusion

A strain of *Saccharomyces cerevisiae *(XR122N) was evolved by continuous exposure to pretreated pine-wood biomass to develop the daughter strain AJP50. Adding a preculture or short adaptation phase of 24 hours in 7% w/v pretreated pine enhanced the performance of the all strains, including AJP50. AJP50 more rapidly fermented pretreated pine-wood biomass at a high solids loading than its parent, or other *Saccharomyces *strains reported in the literature. Growth comparisons between XR122N and AJP50 in a model hydrolysate medium containing inhibitory compounds found in pretreated biomass showed that AJP50 exited lag phase faster under all conditions tested. This ability is due, in part, to AJP50 rapidly converting FF and HMF to their less toxic alcohol derivatives and recovering from ROS damage more quickly than XR122N. Under industrially relevant conditions of 17.5% w/v pretreated pine solids loading, additional evolutionary engineering was required to decrease the pronounced lag phase. Using a combination of adaptation by inoculation first into a fermentation with a solids loading of 7% w/v for 24 hours, followed by a 10% v/v inoculum (approximately equivalent to 1 g/L cell dry weight) into 17.5% w/v solids, the final strain (AJP50) produced ethanol at more than 80% of the maximum theoretical yield after 72 hours of fermentation and reached more than 90% of the maximum theoretical yield after 120 hours of fermentation.

Our results show that that fermentations of pretreated pine containing liquid and solids, including any inhibitory compounds generated during pretreatment, are possible at higher solids loadings than previously reported in the literature. These fermentations used reduced inoculum sizes and had shortened process times, thereby improving the overall economic viability of a pine-to-ethanol conversion process. Results from future studies characterizing the stability of the strain and analyzing the performance under conditions used with industrial processes (for example, after lyophilization) will be important for optimizing use of AJP50 in industrial applications.

## List of abbreviations

ATP: adenosine triphosphate; dcw: cell dry weight; CU: cellobiase units; FF: furfural; FPU: filter paper units; gdw: gram dry weight; HMF: hydroxymethylfurfural; HPLC: high-performance liquid chromatography; SSF: simultaneous saccharification and fermentation; TSB: tryptone soy broth; YPD: yeast-peptone-dextrose; YPD-AI: YPD agar with inhibitors; YPD-BI: YPD broth with inhibitors; WIS: water-insoluble solids.

## Competing interests

*Saccharomyces cerevisiae *strain AJP50 has a patent pending (PCT/US2009/043358).

## Authors' contributions

GMH participated in the design of experiments, collected the data, and helped write the manuscript. JDP participated in the design of experiments, directed technicians in completing experiments, and helped write the manuscript. All authors read and approved the final manuscript.
